# What Patients Want in a Smartphone App That Supports Colonoscopy Preparation: Qualitative Study to Inform a User-Centered Smartphone App

**DOI:** 10.2196/12242

**Published:** 2019-07-02

**Authors:** Maida J Sewitch, Carlo A Fallone, Peter Ghali, Ga Eun Lee

**Affiliations:** 1 Centre for Outcomes Research and Evaluation Research Institute of the McGill University Health Centre Montreal, QC Canada; 2 Department of Medicine McGill University Montreal, QC Canada; 3 Department of Epidemiology, Biostatistics and Occupational Health McGill University Montreal, QC Canada

**Keywords:** colonoscopy, early detection of cancer, mobile health technology, qualitative research, smartphone, user-centered

## Abstract

**Background:**

The preparation for colonoscopy is elaborate and complex. In the context of colorectal cancer screening, up to 11% of patients do not keep their colonoscopy appointments and up to 33% of those attending their appointments have inadequately cleansed bowels that can delay cancer diagnosis and treatment. A smartphone app may be an acceptable and wide-reaching tool to improve patient adherence to colonoscopy.

**Objective:**

The aim of this qualitative study was to employ a user-centered approach to design the content and features of a smartphone app called *colonAPPscopy* to support individuals preparing for their colonoscopy appointments.

**Methods:**

We conducted 2 focus group discussions (FGDs) with gastroenterology patients treated at the McGill University Health Centre in Montreal, Canada. Patients were aged 50 to 75 years, were English- or French-speaking, and had undergone outpatient colonoscopy in the previous 3 months; they did not have inflammatory bowel disease or colorectal cancer. FGDs were 75 to 90 min, conducted by a trained facilitator, and audiotaped. Participants discussed the electronic health support tools they might use to help them prepare for the colonoscopy, the content needed for colonoscopy preparation, and the features that would make the smartphone app useful. Recordings of FGDs were transcribed and analyzed using thematic analysis to identify key user-defined content and features to inform the design of *colonAPPscopy*.

**Results:**

A total of 9 patients (7 male and 2 female) participated in one of 2 FGDs. Main content areas focused on bowel preparation instructions, medication restrictions, appointment logistics, communication, and postcolonoscopy expectations. Design features to make the app useful and engaging included minimization of data input, reminders and alerts for up to 7 days precolonoscopy, and visual aids. Participants wanted a smartphone app that comes from a trusted source, sends timely and tailored messages, provides reassurance, provides clear instructions, and is simple to use.

**Conclusions:**

Participants identified the need for postcolonoscopy information as well as reminders and alerts in the week before colonoscopy, novel content, and features that had not been included in previous smartphone-based strategies for colonoscopy preparation. The ability to tailor instructions made the smartphone app preferable to other modes of delivery. Study findings recognize the importance of including potential users in the development phase of building a smartphone app.

## Introduction

Colorectal cancer screening has been recommended by the Canadian Task Force on Preventive Health Care for individuals aged 50 to 75 years since 2001 [1,2]. Colorectal cancer screening is a preventive health behavior that typically increases the chance of survival by detecting and treating precancerous polyps (growths) and asymptomatic colorectal cancer. Colonoscopy is the recommended follow-up examination for individuals who screen positive to stool testing for colorectal cancer and are at high-risk for colorectal polyps and cancer. However, colonoscopy is an invasive procedure that requires complex and elaborate preparation including consumption of a laxative, restriction of food and liquids, and cessation of certain medications in the week leading up to the colonoscopy appointment. Studies show that up to 11% of individuals do not keep their colonoscopy appointments [3,4] and up to 33% of those attending colonoscopy have inadequately cleansed bowels that can impair colonic visualization [5-11]. Nonadherence to colonoscopy follow-up can delay cancer diagnosis and treatment and waste health care resources in terms of the financial costs for rescheduled and repeat colonoscopies. This is noteworthy because, in Canada, colorectal cancer is the second most commonly diagnosed cancer and the second and third leading causes of cancer deaths in men and women, respectively [12].

Patient educational strategies have been used to improve patient adherence to colonoscopy follow-up. Tools including booklets, cartoons, and short message service (SMS) text messaging have been shown to boost rates of colonoscopy follow-up compared with usual care [13]. More recently, smartphone-based strategies have been developed to help patients prepare for colonoscopy [14-24]. Studies of smartphone-based strategies supporting colonoscopy preparation most often show better outcomes (eg, higher bowel cleansing quality scores) in the intervention groups compared with usual care control groups [14-17,22-24]. However, there was no evidence that the designs of these smartphone-based tools were informed by input from intended end-user groups [14-17,22-24], limiting their utility. Involvement of potential users in the early stages of app development is likely to optimize usage by aligning the app content and features with user-elicited preferences [25,26]. User-centered models involve understanding the environment of intended end users, often through focus group discussions (FGDs) [27]. In fact, one Canadian provincial colorectal cancer screening program that sought to increase screening uptake conducted FGDs to optimize the content and features of its mailed invitations [28]. Feedback obtained from intended users was then used to guide the redesign of the mailed invitations [28].

Mobile health apps have been created following a qualitative user-centered approach in which FGD methodology was used in the design phase to provide insights into end-user needs [29-31]. Previous apps addressed enduring or modifiable health behaviors, such as sun protection, diet, and physical activity [29-31], and their features may not be relevant to screening colonoscopy, a preventive health behavior that occurs once every 10 years according to Canadian guidelines [1,2]. To date, little is known about user-elicited preferences for an app that supports colonoscopy screening.

In this study, we employed a user-centered approach to design a smartphone app called *colonAPPScopy* to support the needs of patients preparing for colonoscopy in the context of colorectal cancer screening. We conducted FGDs to elicit user preferences to inform the content and features of our smartphone app.

## Methods

### Study Design

We conducted FGDs with individuals who recently underwent colonoscopy as they would be knowledgeable about user needs and preferences during colonoscopy preparation. Focus groups were considered the most appropriate method to collect data as group interactions enable the exploration of a broader range of views and prompt group members to frame and clarify individual and shared views [32,33]. The aims of the FGDs were to determine the content (*what*) and features (*how*) of the smartphone app.

### Participants and Recruitment

Gastroenterology outpatients at the McGill University Health Centre in Montreal, Canada, were eligible for participation if they were aged 50 to 75 years, were fluent in English or French, and had undergone colonoscopy in the context of screening within the past 3 months. We excluded patients with inflammatory bowel disease or colorectal cancer as these patients routinely undergo colonoscopy. Given their frequent experiences with colonoscopy preparation, we assumed their needs would be different than the needs of our intended end users who have a screening indication for colonoscopy.

Participants were recruited through purposive sampling. At their colonoscopy appointments, eligible patients were asked by their gastroenterologist if they were interested in participating in a study to build a smartphone app to support patients preparing for colonoscopy. The study research assistant subsequently phoned them to provide further information about the study and invited them to participate in an FGD. Potential participants were made aware of the topic of the focus group at the time they were invited to join, which gave them time to consider what they wanted and did not want to discuss. Participants were remunerated Can $25 for parking or transportation, and a lottery for Can $25 was implemented to encourage participation.

### Data Collection

The FGDs took place in the small conference room of the Division of Clinical Epidemiology at the McGill University Health Centre in April and May 2015. Both FGDs were conducted by the same trained female facilitator. At the beginning of each FGD, the facilitator made participants aware that she was not a part of the clinical care team to reduce the potential for social desirability bias. The facilitator led the FGDs using the focus group guide that was created for the purpose of this study, with the first author (MJS), who was also not a member of the clinical care team, taking field notes.

**Focus group guide.**

Icebreaker questions:

- Do you use the internet to get information on health or health care?
- How helpful is the information you get from the internet?

Key questions:

- When you think about getting health information from the internet, what features do you find attractive/unattractive?
- What information contained in the app would you like to be available to you before you undergo colonoscopy?
- How would you like this information to be available? Prompts: cartoons, figures, web links.
- Are there specific features/tools that would help you follow doctors’ orders for preparing for your colonoscopy?

Discussion topics focused on patients’ experience with using electronic health support tools, the information needed to prepare for colonoscopy, and the preferred format for communicating the information. The focus group questions are presented in Textbox 1. Discussions were 75 to 90 min in duration and audiotaped.

### Data Analysis

Audiotapes of the FGDs were transcribed verbatim by the focus group facilitator. A total of 2 reviewers analyzed the transcripts using thematic analysis, a flexible method for identifying, analyzing, and reporting patterns within data that is not tied to a specific theoretical perspective [34]. The analysis was data driven; the 2 reviewers independently read all transcripts to familiarize themselves with the data and manually recorded their initial codes. Each reviewer then organized and combined recurrent codes into broader themes. Data triangulation was achieved by cross-checking data sources and involving 2 reviewers in the study to discuss discrepancies before agreeing on a final set of themes [35].

### Ethics

Ethics approval was obtained from the Research Ethics Board of the McGill University Hospital Centre (14-084-PSY-T). Written informed consent was obtained from participants at the focus group meetings before beginning the discussions. Audio recordings and transcripts were stored according to policies of the McGill University Hospital Centre.

## Results

### Overview

In total, 12 gastroenterology patients (8 male and 4 female) agreed to join the FGDs. A minimum of 5 participants per focus group was arranged to ensure adequate breadth and depth of discussions [32]. However, on the day of the FGDs, 3 participants did not show; thus, 9 participants (7 male and 2 female) attended one of 2 FGDs. Participants included 4 male and 2 female participants in the first focus group (1 male no-show) and 3 male participants in the second focus group (2 female no-shows). Data saturation was achieved after 2 FGDs.

When participants were asked to discuss the informational or educational content that could be included in the app, 5 content areas emerged: (1) information and instructions for bowel preparation (laxative, diet, and clear liquids), (2) medication restrictions, (3) appointment logistics, (4) communication with endoscopy staff, and (5) what to expect postcolonoscopy. Minimization of data input, reminders and alerts up to 7 days precolonoscopy, and visual aids were identified as possible features of the smartphone app. Discussions were organized into 5 main themes to qualify the content (*what*) and features (*how*) of what users wanted in a smartphone app that supports colonoscopy preparation.

### Trusted Source

As an icebreaker question, participants were asked about the internet, another electronic health tool used frequently by older adults and the colonoscopy screening population. All participants owned smartphones and had used the internet to obtain health-related information. Participants used the internet to obtain health information on colonoscopy and felt there was *no judgement* owing to the anonymity of the internet. However, the anonymity of both users and sources of information as well as the abundance of information on the internet might create a lack of trust:

The internet is dramatic...not possible to tell who to trust.

FGD 1, P1

The internet has too many sources, too much information...exaggerated...what do you trust?

FGD 2, P1

Some participants suggested that the information on the internet was exaggerated, conflicting, and fear-inducing. Accordingly, participants expressed the importance of including the name of the sponsoring institution in the smartphone app to increase user confidence that it comes from a trusted source:

The (name of our institution) should be in the app...to allay fear and find (easily) on searches.

FGD 1, P3

### Timely and Tailored Messages

Some participants needed to stop medications before the colonoscopy appointment but were confused about when and which medications to stop. Participants suggested that the smartphone app should automatically send reminders if someone was taking these medications. Reminders and alerts that are tailored to each patient based on the date and time of the colonoscopy appointment were discussed as an essential feature to the app, as colonoscopy appointments are often scheduled months in advance. Priority alerts included purchasing of the laxative, timing of laxative consumption, timing of medication restrictions, and timing of food and liquid restrictions. Alerts to initiate behaviors were felt to be most effective if accompanied by acoustic signals (eg, beeping for when to purchase the laxative and when to begin the laxative):

...need reminders or you can miss doing the right thing...like stop eating nuts 1 week before.

FGD 2, P1

The app would provide reassurance and confidence that things are done correctly, on time and at the right time.

FGD 1, P3

Moreover, participants indicated that the ability of the smartphone app to tailor messages to individual users made it preferable to other modes of information delivery (eg, paper, Web-based and SMS text messaging) that are less tailored to the user:

Although the internet can provide the same information as the smartphone app, the app may be more helpful because (tailored) alerts can remove fear and (make sure) you take the right thing at the right time.

FGD 2, P3

When prompted for a wish list of features, 1 participant suggested implementing a real-time report on the wait time to colonoscopy that facilitates timing of the drive to the appointment; another participant suggested implementing a real-time report in the endoscopy waiting room.

### Reassurance

When asked to discuss the informational or educational content that could be included in the app, participants emphasized the importance of having an introduction that described the colonoscopy and its purpose. A smartphone app for colonoscopy preparation had the potential to reassure patients that they were doing the right thing at the right time:

I need reassurance I am doing it correctly...had to review the paper (instructions) every hour to be sure I was following instructions.

FGD 2, P3

Some participants noted feeling anxious about administrative and logistical arrangements for their colonoscopy appointments. Noting that hospital cards may have expired by the time of their colonoscopy appointments, participants wanted the smartphone app to provide the location of the office where new cards are issued and provide a message to allow sufficient time to get a new card. Similarly, participants felt the app should provide logistical information for the day of the appointment such as directions to the hospital and the location of the endoscopy unit:

The hardest thing in the day is (knowing) how to get there.

FGD 2, P2

Directions on how to get there (the endoscopy unit) would help relieve stress.

FGD 1, P1

Communication with endoscopy staff through the smartphone app was thought to be important. Participants expressed wanting to speak with a knowledgeable person to ease their concerns regarding their colonoscopy and, more specifically, about adverse events and what to do in case of emergencies:

I’d like to have (had) a contact number for possible complications to know if it’s serious.

FGD 1, P2

Doc or staff should be available for adverse events.

FGD 2, P1

First-time colonoscopy attenders explained that they did not know what to expect or do after the colonoscopy, such as what foods to eat or avoid postcolonoscopy. Some participants wanted information on possible adverse events or complications. They thought the smartphone app could provide the relevant details for users to distinguish minor from serious postcolonoscopy adverse events that required a visit to the hospital. But others thought it was not wise to present this information to all users:

It’s not good to tell anxious people about adverse events...creates fear.

FGD 2, P1

A total of 2 participants suggested the smartphone app could include videos of a colonoscopy being performed as well as testimonials from former patients, but these ideas were not widely endorsed by the group.

### Clear Instructions

Participants wanted information presented in a clear manner at all steps of colonoscopy preparation. Many participants recalled being confused about the consumption of the laxative during the colonoscopy preparation. One participant remembered being confused about the timing of the laxative and had gone shopping before the laxative took effect:

I made a mistake of getting up too quickly and went shopping.

FGD 2, P3

Participants discussed including visual aids (ie, photos) of the different laxatives for easy identification at the time of purchase:

...include photos what to buy (the laxative)...not sure what I was supposed get...photo would help.

FGD 1, P4

Similarly, participants mentioned using colors to signal warnings or restrictions (ie, red) and what was permitted (ie, green) during the colonoscopy preparation:

Warnings should come in a different color...different colored warnings would help to know what and what not to eat or drink.

FGD 1, P1

### Simple to Use

Participants suggested minimizing the amount of data to enter into the app by using check boxes and drop-down menus. These features would also reduce errors and increase user friendliness. Complicated data input (eg, entering the name of a medication) was described as frustrating and time-consuming:

Keep it simple...enter the date of the colonoscopy and the app does the rest.

FGD 1, P4

Make life easy, to help you do the right thing at the right time...just enter the data and the app does the rest.

FGD 1, P2

Participants discussed design features that might complicate and thereby negatively impact the use of the app. These features included cartoons that are demeaning and screens that contain visuals that detract from the message:

Not too busy, not too dark, not too many visuals, not too colorful.

FGD 1, P2

## Discussion

### Principal Findings

Participants used the internet to search for information on colonoscopy preparation but were not satisfied with the colonoscopy information found online, saying it was exaggerated, conflicting, and fear-inducing. Having the information come from a trusted and reliable source such as the user’s health care institution would reassure patients of correctly following the bowel preparation instructions as well as the numerous other behaviors involved in preparing for the colonoscopy appointment. Smartphone apps have the potential to tailor messages and makes them preferable to paper, online, and SMS text messaging modes of information delivery. Participants wanted an app that comes from a trusted source, sends timely and tailored messages, reassures users about all steps in preparing for the colonoscopy appointment, has clear instructions, and is simple to use.

### Comparison With Previous Smartphone-Based Strategies

Our findings are similar to those of other qualitative studies that used focus groups to inform the development of mobile health apps, in that participants wanted the apps to contain clear and concise messages tailored to users [29,31], and for the apps to facilitate communication with clinicians [30]. Our findings are also similar to those of a Canadian study that sought patient input to inform the design of a mailed invitation letter for stool-based testing for colorectal cancer screening, in which key themes were that invitation letters come from a trusted source (patient’s own family physician) and that messages be brief and succinct [28].

In this study, focus group participants identified the same content areas of previously developed smartphone-based strategies for colonoscopy preparation, such as instructions on laxative consumption and restrictions on food, fluid, and medication [14-17,22-24]. However, compared with the general instructions of previously developed colonoscopy apps, participants wanted to input personal data to create automated tailored messages and for the instructions to be clear and simple to follow. Participants also recognized that colonoscopy requires the completion of multiple complex steps in the week leading to the colonoscopy appointment; they wanted the app to send timely reminders to reassure users. Such reminders included alerts for purchasing and ingesting laxatives, medication restrictions tailored to individuals, and administrative and logistical preparation such as renewing hospital cards. Some of these instructions may have been provided at the time of colonoscopy scheduling but are likely to be forgotten when the colonoscopy preparation begins, as the mean wait time from scheduling to undergoing colonoscopy in Canada is 79 (SD 101) days [36]. Reminders may be essential to the effectiveness of a colonoscopy smartphone app; 1 randomized controlled trial found the same quality of bowel cleanliness in the app and control groups when the app was not enabled to send reminders [21].

Personalized reminders and alerts have been shown to increase the uptake of electronic health technologies in modifying various health behaviors [37]. Although SMS text messaging is already well-known and useful for concise time-sensitive information, SMS text messages can contain only 160 characters, limiting the amount of information that can be communicated and personalized to the user. In contrast to SMS text messaging, the content of smartphone app notifications (eg, reminders and alerts) can be more flexible to tailor messages that meet the needs and preferences of the user. Moreover, once the app is set up, notifications are functional without an internet connection, whereas SMS text messaging requires a medically secure server that is expensive and at risk of being hacked. Future studies will determine whether individuals are willing to download an app for single occasion use.

In our study, postcolonoscopy expectations was another content area that was not addressed by previous smartphone apps. Understandably, patients are concerned about food restrictions and adverse events after their colonoscopies. About 25% of patients experience a minor adverse event within 48 hours of a colonoscopy and 0.5% experience a serious adverse event within 30 days [38]. Given the high frequency of adverse events, providing details on the common adverse events and their degrees of severity in the app could result in fewer consultations with health care providers for complications that are self-limited [39]. To reduce arousing fear and anxiety, information on adverse events could be available but not imposed as an app feature.

### Strengths and Limitations

To our knowledge, this is the first study of a smartphone-based strategy for colonoscopy preparation to include potential users at the design phase. We employed a user-centered approach to understand the needs and preferences of patients who had recently undergone colonoscopy in the Canadian context of colorectal cancer screening that begins with stool testing. A second strength was that the 2 focus groups were conducted by the same focus group moderator, under the same conditions, and in the same physical setting to reduce variability in responses owing to environmental factors.

Participants in our study did not represent all patients who undergo colonoscopy; the lack of representativeness is a limitation of all qualitative studies [32]. Focus group participants had undergone colonoscopy and their opinions may not reflect individuals who opted not to have the procedure. Findings may not reflect the needs and preferences of patients who are not fluent in English or French. Further study is needed to determine if the app encourages a larger proportion of individuals to follow through with colonoscopy. Study participation may have been motivated by the desire to make improvements to the delivery of colonoscopy services or by the chance to win the Can $25 lottery. Although the sample size was small, data saturation was reached with both focus groups discussing overlapping topics except for inclusion of videos in the app and for wanting the app to provide real-time updates on time to the next colonoscopy. A recent methodology paper found that greater than 80% of all themes are discoverable within 2 to 3 FGDs [40].

### Development of colonAPPscopy

Our smartphone app, *colonAPPscopy,* has recently been developed, incorporating the insights gained from these FGDs, which identified novel content and features to meet users’ informational and support needs. When designing the content and features of our smartphone app, we attempted to capture the qualities revealed in our FGDs: trusted source, timely and tailored messages, reassurance, clear instructions, and simple to use.

**Figure 1:**
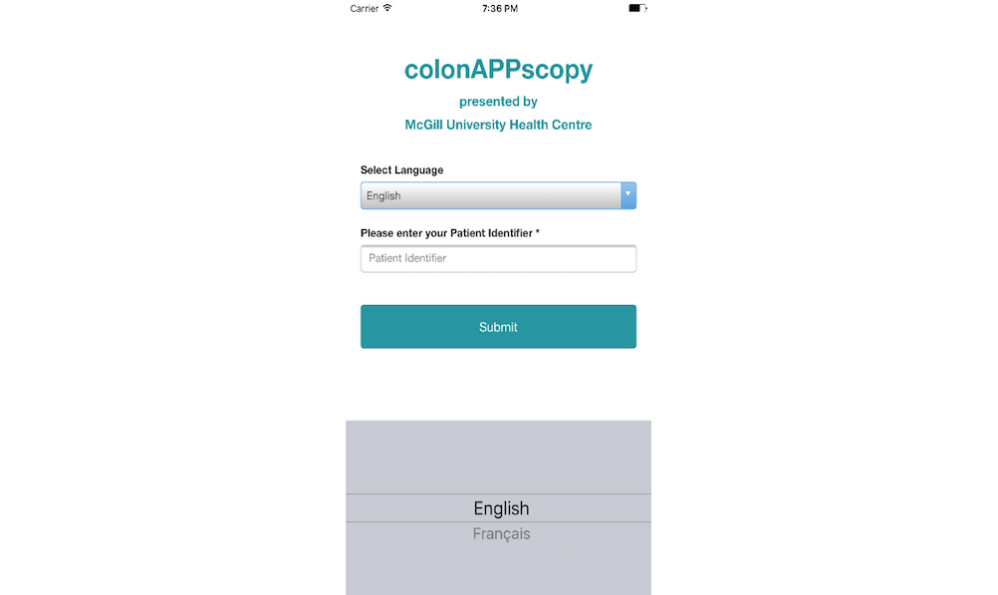
Screenshot of title page of colonAPPscopy.

**Figure 2:**
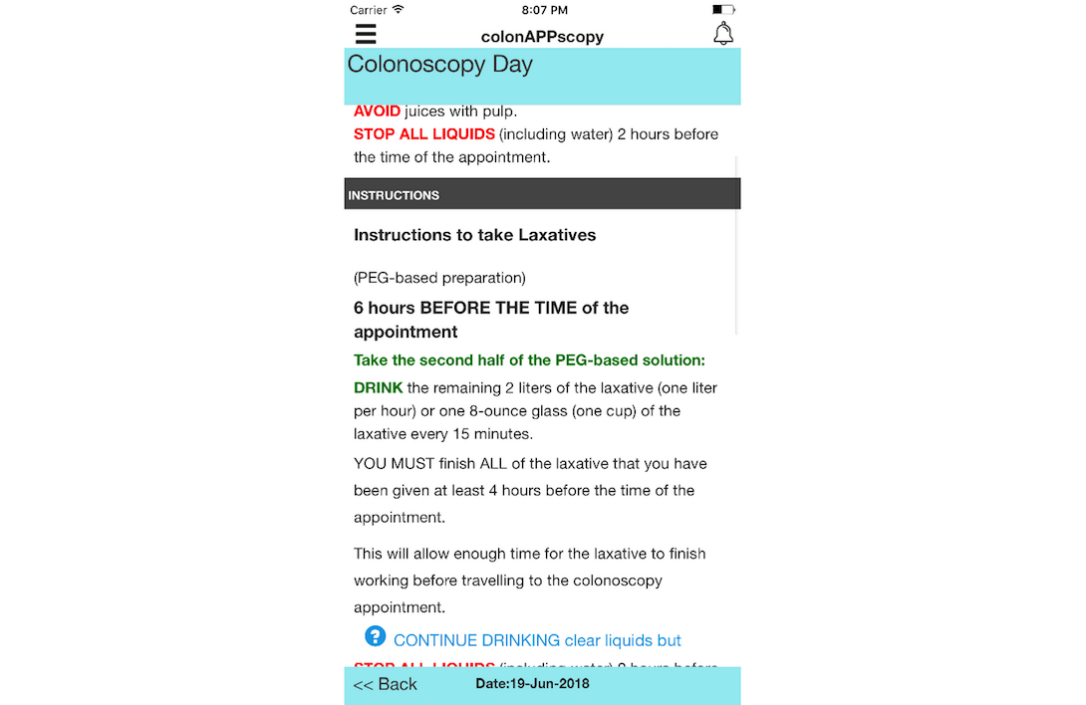
Screenshot of the colonoscopy day in colonAPPscopy.

The title screen of *colonAPPscopy* includes the name of our institution (Figure 1). Content areas include the following: (1) colonoscopy description and purpose, (2) food, liquid, and medication restrictions, (3) logistics of preparation for the colonoscopy appointment, (4) clear liquid diet and laxative explanations and instructions, and (5) what to expect postcolonoscopy including when to seek medical help. Features include the following: (1) check boxes or drop-down menus for simple data input. Data refer to the colonoscopy appointment (eg, appointment time, date, hospital, and gastroenterologist) and to the user’s medical history (medications and diseases); (2) daily tailored reminders and alerts beginning 7 days before colonoscopy for behaviors such as stopping medications, foods, and liquids, beginning the laxative, checking hospital cards are up-to-date, and finding someone to accompany them to the appointment; (3) disability considerations (eg, ability to resize content for users with visual impairments, good color contrast to assist users with cognitive impairments, the appearance of a bell image to indicate notifications for users with hearing impairments, other cues such as capital letters and high contrast for headings, red and green colored letters for important alerts, and blue buttons to immediately gain access to specified information; Figure 2); and (4) safety considerations (each medication that needs to be stopped is accompanied by a red alert instructing the user to consult their physician about the number of days before the colonoscopy that it needs to be stopped, which the user enters into the app, allowing tailored messages to be sent to the user). Features not included in the app prototype are videos owing to budgetary constraints and communications with the endoscopy unit staff owing to firewalls, but direct phone numbers are provided.

Development of *colonAPPscopy* was informed by user-elicited preferences as well as by endoscopist knowledge and experience, and by the core constructs of the Health Belief Model, which was developed specifically to explain preventive health behavior (perceived susceptibility and severity; perceived benefits and barriers; cues to action; and self-efficacy) [41]. Beta testing for comprehension of the English version of the prototype was conducted with 20 patients and then the text was translated into French. We are currently pretesting the bilingual *colonAPPscopy* prototype with users for acceptability and feasibility in a single institution, continuing with the next step of the user-centered design process. For safety reasons, patients are always given both usual care and access to *colonAPPscopy* [42]. Privacy of notifications depends on users securing their smartphones with passwords.

### Conclusions

Findings from this qualitative study were used to guide the development of *colonAPPscopy*, a smartphone app for colonoscopy appointment preparation. Potential end users wanted an app that comes from a trusted source, is capable of sending timely and tailored messages, provides reassurance, has clear instructions, and is simple to use. Including users at the design stage resulted in the identification of content and features not considered in previous similar colonoscopy smartphone apps that were not informed by user perspectives. Our user-centered smartphone app, *colonAPPscopy*, has the potential to be more relevant to the broader patient community and to result in greater uptake, better patient adherence, and improved patient health outcomes.

## Abbreviations

FGD: focus group discussion
SMS: short message service

## References

[1] Canadian Task Force on Preventive Health Care (2001). Colorectal cancer screening. Recommendation statement from the Canadian task force on preventive health care. CMAJ.

[2] Canadian Task Force on Preventive Health Care (2016). Recommendations on screening for colorectal cancer in primary care. CMAJ.

[3] Chopra D, Hookey LC (2016). Comorbid illness, bowel preparation, and logistical constraints are key reasons for outpatient colonoscopy nonattendance. Can J Gastroenterol Hepatol.

[4] Dalton AR (2018). Incomplete diagnostic follow-up after a positive colorectal cancer screening test: a systematic review. J Public Health (Oxf).

[5] Kurlander JE, Sondhi AR, Waljee AK, Menees SB, Connell CM, Schoenfeld PS, Saini SD (2016). How efficacious are patient education interventions to improve bowel preparation for colonoscopy? A systematic review. PLoS One.

[6] Menees SB, Kim HM, Wren P, Zikmund-Fisher BJ, Elta GH, Foster S, Korsnes S, Graustein B, Schoenfeld P (2014). Patient compliance and suboptimal bowel preparation with split-dose bowel regimen in average-risk screening colonoscopy. Gastrointest Endosc.

[7] Kherad O, Restellini S, Martel M, Barkun AN (2015). Polyethylene glycol versus sodium picosulfalte bowel preparation in the setting of a colorectal cancer screening program. Can J Gastroenterol Hepatol.

[8] Dik VK, Moons LM, Hüyük M, van der Schaar P, de Vos Tot Nederveen WH, Ter Borg PC, Meijssen MA, Ouwendijk RJ, Le Fèvre DM, Stouten M, van der Galiën O, Hiemstra TJ, Monkelbaan JF, van Oijen MG, Siersema PD, Colonoscopy Quality Initiative (2015). Predicting inadequate bowel preparation for colonoscopy in participants receiving split-dose bowel preparation: development and validation of a prediction score. Gastrointest Endosc.

[9] Yee R, Manoharan S, Hall C, Hayashi A (2015). Optimizing bowel preparation for colonoscopy: what are the predictors of an inadequate preparation?. Am J Surg.

[10] Govani SM, Elliott EE, Menees SB, Judd SL, Saini SD, Anastassiades CP, Urganus AL, Boyce SJ, Schoenfeld PS (2016). Predictors of suboptimal bowel preparation in asymptomatic patients undergoing average-risk screening colonoscopy. World J Gastrointest Endosc.

[11] Sulz MC, Kröger A, Prakash M, Manser CN, Heinrich H, Misselwitz B (2016). Meta-analysis of the effect of bowel preparation on adenoma detection: early adenomas affected stronger than advanced adenomas. PLoS One.

[12] (2017). Canadian Cancer Society.

[13] Liu Z, Zhang MM, Li YY, Li LX, Li YQ (2017). Enhanced education for bowel preparation before colonoscopy: a state-of-the-art review. J Dig Dis.

[14] Back SY, Kim HG, Ahn EM, Park S, Jeon SR, Im HH, Kim JO, Ko BM, Lee JS, Lee TH, Cho JH (2018). Impact of patient audiovisual re-education via a smartphone on the quality of bowel preparation before colonoscopy: a single-blinded randomized study. Gastrointest Endosc.

[15] Lee YJ, Kim ES, Choi JH, Lee KI, Park KS, Cho KB, Jang BK, Chung WJ, Hwang JS (2015). Impact of reinforced education by telephone and short message service on the quality of bowel preparation: a randomized controlled study. Endoscopy.

[16] Park J, Kim TO, Lee NY, Kim H, Seo EH, Heo NY, Park SH, Moon YS (2015). The effectiveness of short message service to assure the preparation-to-colonoscopy interval before bowel preparation for colonoscopy. Gastroenterol Res Pract.

[17] Kang X, Zhao L, Leung F, Luo H, Wang L, Wu J, Guo X, Wang X, Zhang L, Hui N, Tao Q, Jia H, Liu Z, Chen Z, Liu J, Wu K, Fan D, Pan Y, Guo X (2016). Delivery of instructions via mobile social media app increases quality of bowel preparation. Clin Gastroenterol Hepatol.

[18] Jung JW, Park J, Jeon GJ, Moon YS, Yang SY, Kim TO, Jung ET, Kim HC (2017). The effectiveness of personalized bowel preparation using a smartphone camera application: a randomized pilot study. Gastroenterol Res Pract.

[19] Lorenzo-Zúñiga V, Moreno de Vega V, Marín I, Barberá M, Boix J (2015). Improving the quality of colonoscopy bowel preparation using a smart phone application: a randomized trial. Dig Endosc.

[20] Deng X, Wang Y, Zhu T, Zhang W, Yin Y, Ye L (2015). Short message service (SMS) can enhance compliance and reduce cancellations in a sedation gastrointestinal endoscopy center: a prospective randomized controlled trial. J Med Syst.

[21] Sharara AI, Chalhoub JM, Beydoun M, Shayto RH, Chehab H, Harb AH, Mourad FH, Sarkis FS (2017). A customized mobile application in colonoscopy preparation: a randomized controlled trial. Clin Transl Gastroenterol.

[22] Walter BM, Klare P, Neu B, Schmid RM, von Delius S (2016). Development and testing of an automated 4-day text messaging guidance as an aid for improving colonoscopy preparation. JMIR Mhealth Uhealth.

[23] Walter B, Schmid R, von Delius S (2017). A smartphone app for improvement of colonoscopy preparation (ColoprAPP): development and feasibility study. JMIR Mhealth Uhealth.

[24] Cho J, Lee S, Shin JA, Kim JH, Lee HS (2017). The impact of patient education with a smartphone application on the quality of bowel preparation for screening colonoscopy. Clin Endosc.

[25] Collins LM, Murphy SA, Strecher V (2007). The multiphase optimization strategy (MOST) and the sequential multiple assignment randomized trial (SMART): new methods for more potent eHealth interventions. Am J Prev Med.

[26] van Gemert-Pijnen JE, Nijland N, van Limburg M, Ossebaard HC, Kelders SM, Eysenbach G, Seydel ER (2011). A holistic framework to improve the uptake and impact of eHealth technologies. J Med Internet Res.

[27] Schnall R, Rojas M, Bakken S, Brown W, Carballo-Dieguez A, Carry M, Gelaude D, Mosley JP, Travers J (2016). A user-centered model for designing consumer mobile health (mHealth) applications (apps). J Biomed Inform.

[28] Tinmouth J, Ritvo P, McGregor SE, Claus D, Pasut G, Myers RE, Guglietti C, Paszat LF, Hilsden RJ, Rabeneck L (2011). A qualitative evaluation of strategies to increase colorectal cancer screening uptake. Can Fam Physician.

[29] Buller DB, Berwick M, Shane J, Kane I, Lantz K, Buller MK (2013). User-centered development of a smart phone mobile application delivering personalized real-time advice on sun protection. Transl Behav Med.

[30] Robertson MC, Tsai E, Lyons EJ, Srinivasan S, Swartz MC, Baum ML, Basen-Engquist KM (2017). Mobile health physical activity intervention preferences in cancer survivors: a qualitative study. JMIR Mhealth Uhealth.

[31] Henry BL, Quintana E, Moore DJ, Garcia J, Montoya JL (2019). Focus groups inform a mobile health intervention to promote adherence to a Mediterranean diet and engagement in physical activity among people living with HIV. BMC Public Health.

[32] Kitzinger J (1995). Qualitative research. Introducing focus groups. Br Med J.

[33] Tong A, Sainsbury P, Craig J (2007). Consolidated criteria for reporting qualitative research (COREQ): a 32-item checklist for interviews and focus groups. Int J Qual Health Care.

[34] Braun V, Clarke V (2006). Using thematic analysis in psychology. Qual Res Psychol.

[35] Carter N, Bryant-Lukosius D, DiCenso A, Blythe J, Neville AJ (2014). The use of triangulation in qualitative research. Oncol Nurs Forum.

[36] Sey MS, Gregor J, Adams P, Khanna N, Vinden C, Driman D, Chande N (2012). Wait times for diagnostic colonoscopy among outpatients with colorectal cancer: a comparison with Canadian Association of Gastroenterology targets. Can J Gastroenterol.

[37] Marcolino MS, Oliveira JA, D'Agostino M, Ribeiro AL, Alkmim MB, Novillo-Ortiz D (2018). The impact of mHealth interventions: systematic review of systematic reviews. JMIR Mhealth Uhealth.

[38] Sewitch MJ, Azalgara VM, Sing MF (2018). Screening indication associated with lower likelihood of minor adverse events in patients undergoing outpatient colonoscopy. Gastroenterol Nurs.

[39] Marquez Azalgara V, Sewitch MJ, Joseph L, Barkun AN (2014). Rates of minor adverse events and health resource utilization postcolonoscopy. Can J Gastroenterol Hepatol.

[40] Guest G, Namey E, McKenna K (2016). How many focus groups are enough? Building an evidence base for nonprobability sample sizes. Field Methods.

[41] Rosenstock IM (1974). The health belief model and preventive health behavior. Health Educ Behav.

[42] Bates DW, Landman A, Levine DM (2018). Health apps and health policy: what is needed?. J Am Med Assoc.

